# Human resource development in supply chain management- what do the UN agencies say?

**DOI:** 10.1186/2052-3211-7-S1-P7

**Published:** 2014-12-17

**Authors:** Amrita Sankaranarayanan, Janine Marie Traulsen, Sofia Kälvemark Sporrong, Andrew Brown

**Affiliations:** 1 Institute for Pharmacy, University of Copenhagen, Copenhagen, Denmark; 2 People that Deliver, Copenhagen, Denmark

## Background

Efficient and well trained health workers are required for successful functioning of a supply chain that ensures equitable access to health commodities and universal coverage. Various UN organizations have identified this need as referenced to in a variety of online UN resources. This research aims to collect and analyse this extensive literature systematically and provide evidence for core strengthening parameters of human resources.

## Method

This research employed a "Realist Review" methodology involving a systematic search of the literature in the publicly available websites of UNICEF, UNFPA, WHO and People that Deliver. These documents and reports were then subjected to manual thematic analysis and common themes emerging were extracted and analysed.

## Results

A total of 707 documents underwent initial title screening, with 379 retained. These articles were then subjected to executive summary screening with 182 documents retained. Finally, these remaining documents were retrieved in full, read and a total of 128 documents were retained to undergo thematic analysis. This broad thematic analysis led to the extraction of the following five themes: engage stakeholders, optimize policies and plans, workforce development, increase performance, and professionalize supply chain management. Most of this evidence was pertaining to optimizing policies and plans (48 documents), with the theme of professionalizing supply chain management having the least amount of evidence (4 documents).

## Discussion

The five themes generated from this research are similar to those documented in the USAID Report on Human Resource Capacity Development in Public Health Supply Chain Management and the Human Resources for Health Action Framework- Technical Brief 12. This review synthesizes the UN evidence supporting the importance of these five themes in human resources for health supply chains. Strengthening of these five core parameters as suggested in the above mentioned documents and by the UN agencies is important to ensure sustainable human resources development in this sector. Governments seeking to strengthen their health supply chain systems should consider building up on these five competencies for an effect human resource development.

## Lessons learned

The reports and publications by the UN agencies are a rich source of expert information that should be considered for relevant knowledge synthesis. The five core parameters as found in this research, form a set of building blocks to consider HR for SCM in a systematic way. More evidence needs to be generated to support the professionalizing aspect.

